# Loss of Unconventional Myosin VI Affects cAMP/PKA Signaling in Hindlimb Skeletal Muscle in an Age-Dependent Manner

**DOI:** 10.3389/fphys.2022.933963

**Published:** 2022-06-28

**Authors:** Lilya Lehka, Dominika Wojton, Małgorzata Topolewska, Vira Chumak, Łukasz Majewski, Maria Jolanta Rędowicz

**Affiliations:** ^1^ Laboratory of Molecular Basis of Cell Motility, Nencki Institute of Experimental Biology, Polish Academy of Sciences, Warsaw, Poland; ^2^ Laboratory of Neurodegeneration, International Institute of Molecular and Cell Biology, Warsaw, Poland

**Keywords:** unconventional myosin VI, PKA, CREB, AKAP9, cAMP, Snell’s waltzer mice

## Abstract

Myosin VI (MVI) is a unique unconventional myosin ubiquitously expressed in metazoans. Its diverse cellular functions are mediated by interactions with a number of binding partners present in multi-protein complexes. MVI is proposed to play important roles in muscle function and myogenesis. Previously, we showed that MVI is present in striated muscles and myogenic cells, and MVI interacts with A-kinase anchoring protein 9 (AKAP9), a scaffold for PKA and its regulatory proteins. Since PKA directly phosphorylates the MVI cargo binding domain, we hypothesized that the cellular effects of MVI are mediated by the cAMP/PKA signaling pathway, known to play important roles in skeletal muscle metabolism and myogenesis. To elucidate the potential role of MVI in PKA signaling in hindlimb muscle function, we used mice lacking MVI (Snell’s waltzer, *SV*), considered as natural MVI knockouts, and heterozygous littermates. We used muscles isolated from newborn (P0) as well as 3- and 12-month-old adult mice. We observed a significant increase in the muscle to body mass ratio, which was most evident for the soleus muscle, as well as changes in fiber size, indicating alterations in muscle metabolism. These observations were accompanied by age-dependent changes in the activity of PKA and cAMP/PKA-dependent transcriptional factor (CREB). Additionally, the levels of adenylate cyclase isoforms and phosphodiesterase (PDE4) were age-dependent. Also, cAMP levels were decreased in the muscle of P0 mice. Together, these observations indicate that lack of MVI impairs PKA signaling and results in the observed alterations in the *SV* muscle metabolism, in particular in newborn mice.

## Introduction

**FIGURE 1:**
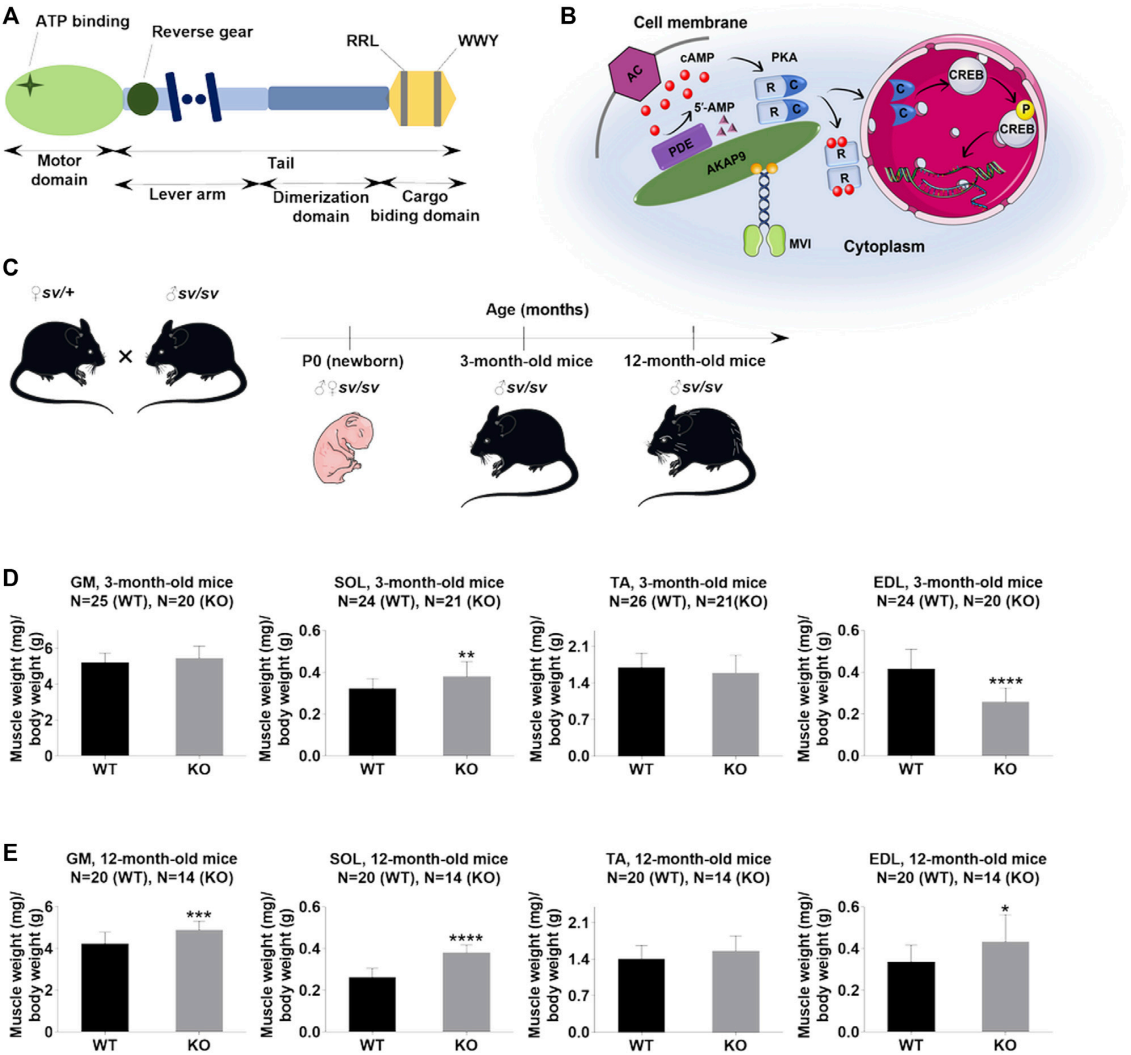
Lack of myosin VI affects the muscle/body mass ratio. **(A)**, Schematic diagram of the organization of the MVI heavy chain; detailed explanation in the text. **(B)**, Schematic representation of the possible role of MVI in cAMP signaling. cAMP produced by adenylate cyclase (AC) binds to cAMP-dependent protein kinase PKA regulatory subunits (R), allowing dissociation of PKA catalytic subunits (c), which phosphorylate target proteins, including CREB. PDE hydrolyzes cAMP to 5′-AMP. A-kinase anchoring protein 9 (AKAP9) organizes PKA signaling components into macromolecular complexes at specific subcellular sites. MVI binds to AKAP9 and is proposed to be involved in transporting the complex to the appropriate destination. **(C)**, Mouse experimental design. **(D)** and **(E)**, Analysis of the muscle/body weight ratio of hindlimb muscles of 3-months- and 12-month-old mice, respectively. GM, gastrocnemius medialis muscle; SOL, soleus muscle; TA, tibialis anterior muscle; EDL, extensor digitorum longus muscle. Data are expressed as mean ± SD, t-test, *p ≤ 0.05, **p ≤ 0.01, ***p ≤ 0.001, ****p ≤ 0.0001; vs. control. **(B)** and **(C)** were created using Servier Medical Art - (smart.servier.com).

Unconventional myosin VI (MVI) is a unique actin-based motor, which unlike other known myosins moves backwards, i.e. towards the minus end of actin filaments (Wells et al., 1999; Elting et al., 2011). It is ubiquitously expressed in all *Metazoans* (Berg et al., 2001). We have shown previously that MVI is present in skeletal muscle where it localizes to the sarcoplasmic reticulum, the postsynaptic region of the neuromuscular junction, and myonuclei (Karolczak et al., 2013; Karolczak et al., 2014). Its ∼140 kDa heavy chain is encoded in humans by a single *MY O 6* gene (Hasson et al., 1996). Similar to other members of the large myosin superfamily, MVI contains the N-terminal motor domain (with the actin and ATP binding sites), a neck region (where two calmodulin molecules bind), and a C-terminal tail (containing the helical and cargo binding domains; Figure 1A) (Hasson and Mooseker, 1994; Hasson et al., 1996). It exists as a monomer or a dimer, and dimerization (occurring *via* a helical region within the tail) is thought to depend on several factors such as cargo binding, monomer availability, and/or phosphorylation within the tail domain (Buss et al., 1998; Yu et al., 2009). MVI is engaged in numerous cellular processes through its interaction with actin (*via* the N-terminal motor domain) and tissue-specific partner proteins binding to the C-terminal cargo domain. Partner recognition occurs either through a positively charged RRL region or a hydrophobic WWY region (Tumbarello et al., 2012; Magistrati and Polo, 2021). Also, a positively charged cluster located within the cargo domain was shown to bind to PIP2-containing liposomes, possibly aiding in the partner binding (Spudich et al., 2007). Numerous tissue-specific MVI-binding-partners have been already identified in mammals, among them are proteins engaged in cytoskeletal dynamics, proteins associated with the Golgi apparatus and endoplasmic reticulum as well as proteins involved in endocytosis and cell adhesion (Karolczak et al., 2013; Tumbarello et al., 2013; Karolczak et al., 2015; Wollscheid et al., 2016; Majewski et al., 2018; Magistrati and Polo, 2021).

Our studies on muscle and myogenic cells have identified several muscle-specific MVI-binding partners, which include A Kinase Anchoring Protein 9 (AKAP9), a regulator of PKA activity (Diviani and Scott, 2001; Ruehr et al., 2003; Taylor et al., 2013; Karolczak et al., 2015). Members of the AKAP family function through the creation of a compartmentalized environment inside the cell to bring PKA signaling molecules to their targets (Figure 1B) (Feliciello et al., 1997; Edwards and Scott, 2000). There are also reports that AKAP9 by targeting PKA to the cytoskeleton could play an important role in the cytoskeleton dynamics (Diviani et al., 2016). It is well known that cAMP/PKA signaling plays important functions in skeletal muscle by its involvement in the metabolism of glucose, glucagon, and lipids necessary for energy production, so crucial for muscles that are high energy consumers (Taylor et al., 2013). Several data associate PKA with muscle function and dysfunction (Morita et al., 1995; Ruehr et al., 2003; Rudolf et al., 2013; Cruz et al., 2018). The observed up-regulation of PKA RI alpha subunit mRNA observed in rat skeletal muscle after nerve injury could be due to the presence of both PKA and AKAP9 within and around the neuromuscular junction (Morita et al., 1995; Perkins et al., 2001). It is noteworthy that another unconventional myosin, myosin V (MV), was found to be involved in the PKA-dependent organization of acetylcholine receptors at the neuromuscular junction (Röder et al., 2010, 2012). Furthermore, it was shown that targeting PKA by AKAP9 regulated phosphorylation and the function of the skeletal muscle ryanodine receptor (Ruehr et al., 2003). Also, it was demonstrated that the activated catalytic subunit of PKA is translocated to the nucleus where it activates CREB (transcription factor with the cAMP response element) thus altering the transcription and therefore synthesis of numerous proteins (Figure 1B) (Altarejos and Montminy, 2011). CREB, mostly known for its function in the central nervous system, has also an important function in skeletal muscle (Carlezon et al., 2005; Altarejos and Montminy, 2011). For example, transgenic expression of dominant-negative A-CREB in skeletal muscle led to a dystrophic phenotype characterized by progressive muscle wasting, muscle inflammation, and myonecrosis (Berdeaux et al., 2007). CREB was also found to increase mitochondrial oxidative capacity in muscle by up-regulating the expression of the nuclear hormone receptor co-activator PGC1α and mitochondria biogenesis during myogenesis (Wu et al., 2006; Franko et al., 2008).

We have shown previously *in vitro* that the C-terminal cargo domain of MVI is phosphorylated directly by PKA. Furthermore, we showed that depletion of MVI in myogenic cells affected levels of cAMP and PKA phosphorylation as well as the level and localization of AKAP9 (Karolczak et al., 2015). These findings suggest that MVI could be involved in PKA signaling in skeletal muscle. To test this hypothesis, we performed studies using hindlimb muscles isolated from newborn (P0) and adult (3- and 12-month-old) Snell’s waltzer (*SV*) mice (Deol and Green, 1966; Avraham et al., 1995). These mice are considered natural MVI knockouts and are a commonly used model in studies on the functions of this molecular motor. Our data provide evidence that MVI is involved in cAMP/PKA signaling, and effects associated with loss of MVI are age-dependent.

## Materials and Methods

### Animals

Hindlimb muscles were derived from Snell’s waltzer (*SV*) mice, natural MVI knockouts (MVI-KO), with a spontaneous intragenic deletion in *Myo6* gene. There is an ongoing colony of these animals that was kindly donated by Dr. Folma Buss from the University of Cambridge. Heterozygous animals (*sv*/+) served as a control group as it has been shown that the presence of one unmutated allele is sufficient to fulfill the MVI functions (Hegan et al., 2015; Marcotti et al., 2016). Analyses were performed on the muscles isolated from mice at different stages of development: newborn mice (P0), 3- and 12-months-old (Figure 1C). In the case of P0, whole leg muscles were used. Animal housing and sacrifice procedures were performed in compliance with the European Communities Council directives adopted by the Polish Parliament (Act of 15 January 2015, on the use of animals in scientific investigations), and work with the mice tissues got approval from the Director of the Nencki Institute of Experimental Biology (approval number: 410/2021/IBD).

### Cell Culture

C2C12 mouse myoblasts (American Type Culture Collection, United States) were maintained in DMEM (31966021, Gibco) containing 4.5 g/L glucose supplemented with GlutaMAX-1, 10% heat-inactivated fetal bovine serum (FBS, 10500064, Gibco) and antibiotics, 1% penicillin/streptomycin (p/s, 15140122, Gibco) at 37°C in humidified air containing 5% CO2. After cells reached the confluence (considered as day 0) the process of differentiation was induced by transferring cells to a medium containing 2% horse serum (HS, 26050088, Gibco) instead of 10% FBS. The culture was continued for up to the next 7–10 days.

### Antibodies

The antibodies were used as follows: mouse monoclonal antibody to glyceraldehyde-3-phosphate dehydrogenase (GAPDH, MAB374, Merck, 1:10000); rabbit polyclonal phosphorylated form of cAMP-dependent protein kinase (pPKA (Thr197), 44988A, Thermo Fisher Scientific, 1:1000); rabbit polyclonal to the recombinant fragment corresponding to a region within amino acids 1 and 351 of PKA catalytic subunit alpha (PA5-21842, Invitrogen, 1:1000); mouse monoclonal to phosphorylated form of cAMP response element-binding protein (CREB (Ser 133), 9196S, Cell Signaling, 1:500); rabbit monoclonal to cAMP response element-binding protein (CREB, 9197S, Cell Signaling, 1:1000); rabbit polyclonal to adenylate cyclase 3 (AC3, PA1-31191, Invitrogen, 1:250); rabbit polyclonal to adenylate cyclase 7 (AC7, PA5-103390, Thermo Fisher Scientific, 1:500); mouse monoclonal to A-kinase anchoring protein 9 (AKAP9, ab32679, Abcam, 1:125); goat monoclonal to A-kinase anchoring protein 9 (AKAP9, ab31307, Abcam, 1:200); rabbit monoclonal to cAMP-specific 3′,5′-cyclic phosphodiesterase 4D (PDE4D, ab171750, Abcam, 1:1000); HRP rabbit polyclonal to *β*-tubulin (ab21058, Abcam, 1:10000); rabbit polyclonal to MVI (25–6791, *Proteus*, 1:500); goat anti-mouse IgG antibody, HRP conjugate (AP308P, Millipore, 1:10000); goat anti-rabbit IgG antibody, HRP conjugate (AP307P, Millipore, 1:10000).

### Subcellular Fractionation

Cytosolic and nuclear fractions of skeletal muscle were obtained using the protocol described in (Dimauro et al., 2012). Briefly, fresh hindlimb muscles were isolated from P0 mice followed by homogenization in 300 µL of buffer 1 containing 250 mM sucrose (S0389-500G, Sigma-Aldrich), 50 mM Tris-HCl pH 7.4, 5 mM MgCl2 (63072, Fluka Chemicals), protease (04693116001, Roche), and phosphatase inhibitor cocktails (0490683700, Roche). After that, the homogenates were maintained on ice, vortexed, and centrifuged for 15 min at 800 × g. Next, the obtained pellet (containing nuclei and cell debris) was kept on ice while the supernatant S was used for the isolation of cytosolic fractions. The pellet was resuspended in 300 µL of buffer 1, vortexed followed by centrifugation at 500 × *g* for 15 min. Next, the nuclear pellet, labeled as P, was kept on ice, while the supernatant was discarded. In order to increase the purity of P fraction, the pellet additionally was washed using buffer 1 (300 µL), vortexed, and then centrifugated at 1000 × g for 15 min. The obtained pellet was further resuspended in 200 µL of buffer 2 consisting of 20 mM HEPES pH 7.9 (H3375-500G, Sigma-Aldrich), 1.5 mM MgCl2, 0.5 M NaCl, 0.2 mM EDTA (EDS-500G, Sigma), 20% glycerol, and 1% Triton X-100 supplemented by phosphatase and protease inhibitors. The pellet was vortexed and incubated on ice for 30 min. The nuclei were lysed using 10 passages through an 18-gauge needle followed by centrifugation at 9000 × *g* for 30 min at 4°C. The supernatant obtained after centrifugation was the final nuclear fraction. The supernatant S was centrifuged at 800 × g for 10 min. After centrifugation, the supernatant S1 was next centrifuged at 11000 × g for 10 min. After that, supernatant S2 containing cytosol fraction was precipitated for 1 h in cold 100% acetone at −20°C. After 5-min centrifugation at 12000 *g*, the pellet was resuspended in 100 µL of buffer 1 and labeled as the cytosolic fraction. The protein content of each fraction was measured using BCA protein assay (23227, Thermo Fisher Scientific).

### Western Blot Analysis

Muscles were homogenized in Bioeko Pro 200 Double insulated tissue homogenizer in 10 volumes of the ice-cold buffer containing 50 mM Tris-HCl pH 7.5 (T6687, Sigma-Aldrich), 150 mM NaCl (117941206, Chempur), 0.5% Triton X-100 (SLCJ7494, Sigma-Aldrich), 5 mM EDTA pH 7.4 (EDS-500G, Sigma-Aldrich), 5% glycerol (114433204, Chempur), 50 mM NaF (71518, Fluka Chemicals), 1 mM Na3VO4 (S6508-50G, Sigma-Aldrich), supplemented by phosphatase and protease inhibitors and boiled in SDS loading buffer. Muscle tissue homogenates (10–30 µg of protein per well) were separated using 8, 10, or 12% polyacrylamide SDS-gels and then transferred to a nitrocellulose membrane (10600002, Amersham). After the transfer, the membrane was blocked for 1 h at room temperature in Tris-buffered saline (TBS) containing 5% non-fat milk powder or 5% BSA (A7906-150G, Sigma-Aldrich) and 0.2% Triton X-100 followed by overnight incubation with appropriate dilutions of primary antibodies. The primary antibodies were detected using 1:10000 dilutions of anti-rabbit, anti-mouse, or anti-goat antibodies conjugated with horseradish peroxidase. The reaction was developed using ECL according to the manufacturer’s instructions (WBKLS0500, Merck).

### Muscle Morphology Analysis

After dissection, the muscles were weighed (Supplementary Table S1) and immediately mounted in an optimal cutting temperature compound (6769006, Thermo Fisher Scientific) and frozen in isopentane, previously cooled in liquid nitrogen. Cryo-sectioning was performed on a Leica cryostat to generate 10 µm sections. After that, muscle cross-sections were air-dried for 30 min and stained with Mayer’s Hematoxylin Solution (MAS16-500ML, Sigma-Aldrich) for 30 min. After rinsing with tap water for 15 min, the sections were stained with Eosin Y Solution Counterstain (HT110216-500ML, Sigma-Aldrich) for 7 min followed by dehydration through 2 changes each of 95% reagent alcohol and xylene for 2 min. After that, the sections were mounted with a resinous mounting medium (HX74511279, Merck). Morphometric analysis was performed on digital muscle cross-sections taken by Nikon Eclipse Ti-U microscope equipped with Nikon Digital Sight DS-U3 camera (Nikon Corporation, Shinagawa, Tokyo, Japan). Analysis of muscle cross-section area, percentage of fibers with appropriate diameter, number of fibers per selected area as well as a number of nuclei per myofiber was carried out on 3–5 sections from three animals in each group, using ImageJ software (Schindelin et al., 2012).

### Evaluation of cAMP Levels

Muscles isolated from the hindlimb of mice of different ages were washed in 1X PBS (MS00YT1001, Biowest), homogenized in 10 volumes of 0.1 M HCl, and centrifuged for 10 min at 600 × g to get rid of cell debris. Intracellular cAMP level in the final supernatants was measured by using a direct cAMP ELISA kit (ADI-900–066, Enzo) according to the manufacturer’s instructions for acetylation format. The protein concentration of the same samples was determined by the Bradford protein quantification assay (500–0205, Bio-Rad). Data were normalized to the protein content.

### Quantitative Real-Time Polymerase Chain Reaction (qRT-PCR)

RNA from hindlimb muscles of newborn mice was isolated using the RNeasy Plus Universal Mini Kit (73404,QIAGEN) according to the manufacturer’s instruction. Removing DNA contamination from RNA samples was performed by treatment with RNase-Free DNase I (ENO521, Thermo Fisher Scientific). First-strand cDNA synthesis was performed using 1 µg of RNA and High-Capacity cDNA Reverse Transcription Kit (4368814, Thermo Fisher Scientific). qPCR reactions were assembled on StepOne Plus Real-Time PCR System (Thermo Fisher Scientific) using SYBR Green master mix (4472908, Thermo Fisher Scientific) along with 2.5 ng of cDNA and 500 nM of primers (Genomed). The mRNA levels were determined using the 2−ΔΔCT method (Livak and Schmittgen, 2001) and target genes were normalized to the housekeeping gene *β*-2 microglobulin (*B2m*). The primers for qPCR were designed using primer-BLAST: for *Prkaca* GAA​AAT​CGT​CTC​TGG​GAA​GGT (forward) and TGG​CAA​TCC​AGT​CAG​TCG​T (reversed); for *Creb1* CAA​GCT​TGT​AAT​GCT​TAG​CAA​CAG (forward) and GGG​CAT​GCA​CAC​GTC​TTA​AC (reversed); for *Akap9* TAA​ACA​GCG​AGA​CGG​CAT​CA (forward) and CGG​CTG​AGA​GTG​CTG​TTT​TG (reversed); for *Adcy2* GAC​TTC​TGC​TTT​CCC​TGC​TG (forward) and TAT​GGC​TTC​GCA​CAT​ATC​CA (reversed); for *Adcy3* TAC​CCA​GCT​GTC​CTC​TGC​TT (forward) and CGT​ACG​AGA​TGG​CCT​CTA​CC (reversed); for *Adcy7* ACT​CTG​GGT​GTG​TCC​TTT​GG (forward) and GCT​TTG​TGC​ATC​AGA​CAG​GA (reversed); and for *B2m* CAT​GGC​TCG​CTC​GGT​GAC​C (forward) and AAT​GTG​AGG​CGG​GTG​GAA​CTG (reversed).

### Immunofluorescence Staining

Hindlimb muscle cross-sections of newborn animals were air-dried for 30 min and were rinsed with 1X PBS for another 15 min. After rinsing, the sections were put in 4% PFA for 30 min. After three wash steps with 1X PBS for 5 min, the sections were incubated with NH4Cl for 30 min followed by blocking in 1X PBS with 0.3% Triton X-100 and 5% horse serum (26050088, Gibco) at 4°C for 1 h. Next, the sections were incubated overnight with antibodies against AKAP9 (ab 31307, Abcam, 1:50) prepared in a blocking solution at 1:125 dilution. Following incubation with the primary antibodies and after rinsing 3 times with 1X PBS with 0.3% Triton X-100, the sections were incubated in blocking solution at room temperature with Alexa Fluor 555-conjugated secondary antibodies to detect primary antibodies (A21432, Invitrogen, 1:1000). Finally, the sections were washed 3 times in 1X PBS with 0.3% Triton X-100 and mounted using Vectashield Plus antifade mounting medium with DAPI (H-2000, Vector Laboratories).

### Identification of the MVI Interaction Site With AKAP9

For this, we used GFP-fused human myosin VI tail containing the large insert (WT, aa 840–1284) and its mutants RRL to AAA (aa 1115–1117) and WWY to WLY (aa 1191–1193) that were generated as previously described in (Arden et al., 2007; Chibalina et al., 2007). The plasmids were a gift from Dr. Folma Buss from Cambridge Institute for Medical Research, UK. C2C12 myoblasts were plated on coverslips in 35-mm dishes, cultured for 24 h, and then transfected with the plasmids encoding GFP-tagged MVI tail fragments using Lipofectamine 3000 according to the manufacturer’s protocol (L3000015, Thermo Fisher Scientific). After transfection, the cells were fixed and immunostained with anti-AKAP9 antibody. Briefly, cells on the coverslips were washed twice with PBS and fixed in 4% paraformaldehyde solution in PBS at 4°C for 20 min. Fixed cells were washed twice with PBS, quenched for 30 min in PBS with 50 mM NH4Cl, and incubated for 1 h in blocking solution (PBS with 2% horse serum and 0.02% Triton-X-100). Coverslips were then incubated for 2 h at room temperature or overnight at 4°C with anti-AKAP9 antibody diluted at 1:50 and then extensively washed. After 1-hour incubation with donkey anti-goat Alexa Fluor® 555 (A21432, Invitrogen, 1:200), coverslips were exhaustively washed and mounted using Vectashield Medium. The images were attained with ZEISS LSM780 spectral confocal microscope equipped with a Plan Apochromat 63x/1.4 Oil DIC lens. Special care was taken to control for any possible cross-reactivity (cross-bleeding) of the detection systems. Co-localization analysis was performed using the Coloc module of the Imaris 8.3 software according to the manufacturer’s instructions (URL: http://www.bitplane.com/imaris/imaris). Analysis was performed on raw data using cytoplasmic regions of interest (ROIs).

### Statistics

Each reported value represents the mean ± S.D. of more than two independent replicates of each of at least three independent experiments. Quantitative data were analyzed by unpaired two-tailed Student’s t-test. All statistical analyses were performed using GraphPad Prism 8.4.3 Software (GraphPad San Diego, CA, United States). *p* < 0.05 was considered statistically significant. *p* < 0.05 was marked as ∗, *p* ≤ 0.01 was marked as ∗∗, *p* ≤ 0.001 was marked as ∗∗∗, and *p* ≤ 0.0001 as ∗∗∗∗.

## Results

The studies were performed using hindlimb skeletal muscles of P0 and adult (3- and 12-month-old) male *SV* mice (Figure 1C). The muscles isolated from the heterozygous littermates were used as controls. The samples derived from *SV* mice are termed KO, and from controls as WT.

### Loss of MVI Affects Muscle and Fiber Size

Adult *SV* animals are smaller than wildtype littermates (Hegan et al., 2015). Our data confirm this finding and additionally show that muscle mass decrease also is found in newborn (P0) mice (Supplementary Figure S1). At 3- and 12-months time points we found changes in muscle/body mass ratio (in mg/g) for gastrocnemius medialis (GM), soleus (SOL), tibialis anterior (TA) and extensor digitorum longus (EDL muscles. As shown in Figure 1D, in 3-month-old mice muscle/body mass ratio was slightly increased in GM, significantly increased in SOL, not affected in TA, and significantly decreased in EDL. In 12-month-old mice, this parameter was increased for all four muscle types with statistical significance for GM, SOL, and EDL muscles (Figure 1E). These data indicate that despite overall mass loss in SV mice, the hindlimb muscles examined here (excluding EDL in 3-months mice) are relatively larger than the muscles of WT littermates.

**FIGURE 2:**
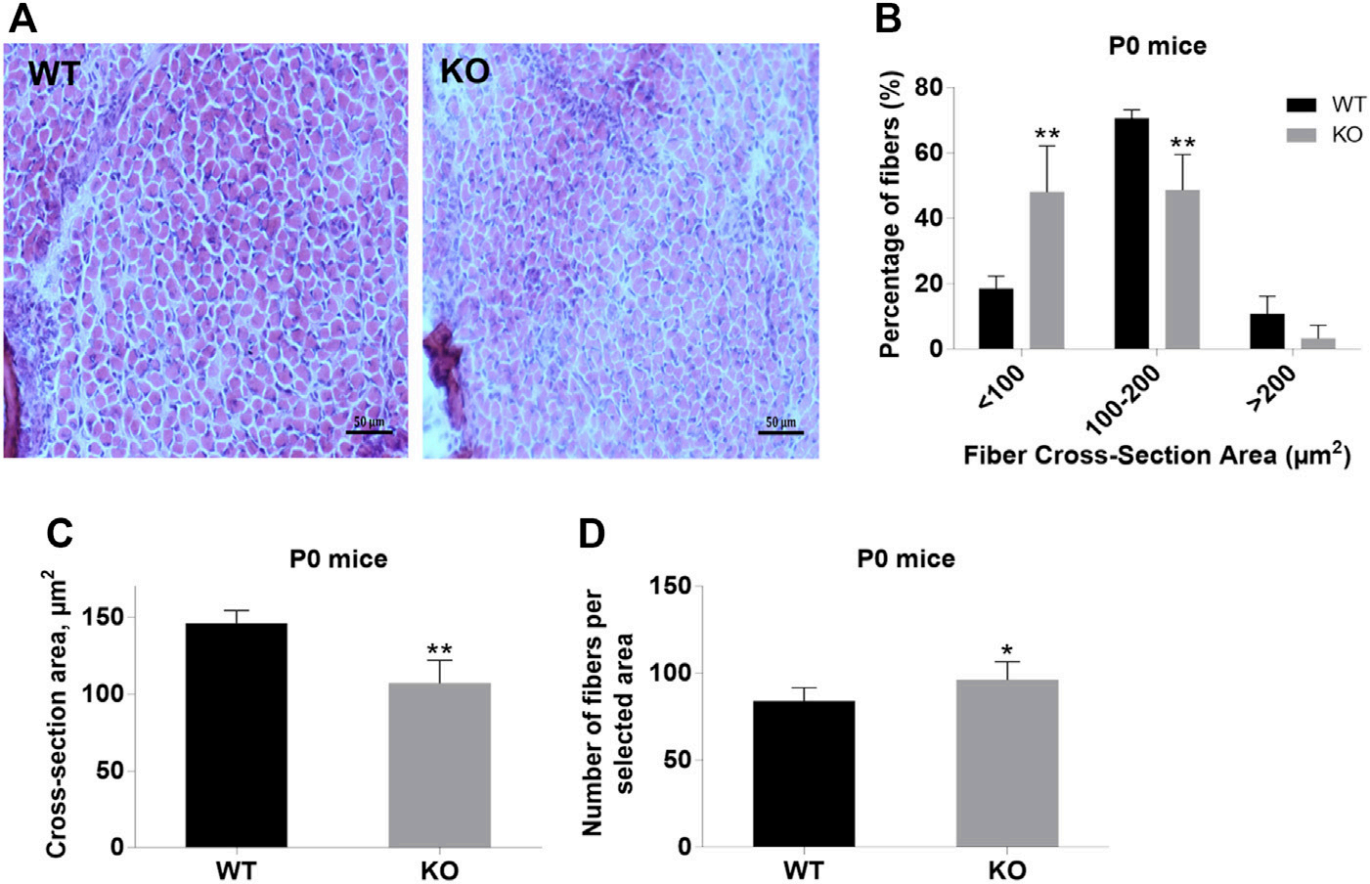
Analysis of hindlimb muscles of newborn (P0) WT and KO mice. **(A)**, Hematoxylin/eosin staining of hindlimb muscles. **(B)**, Size distribution of muscle fibers. **(C)**, Cross-section area of hindlimb muscles. **(D)**, Number of fibers per selected area. Data are expressed as mean ± SD, t-test, **p ≤ 0.01; *p ≤ 0.05 vs. control.

**FIGURE 3:**
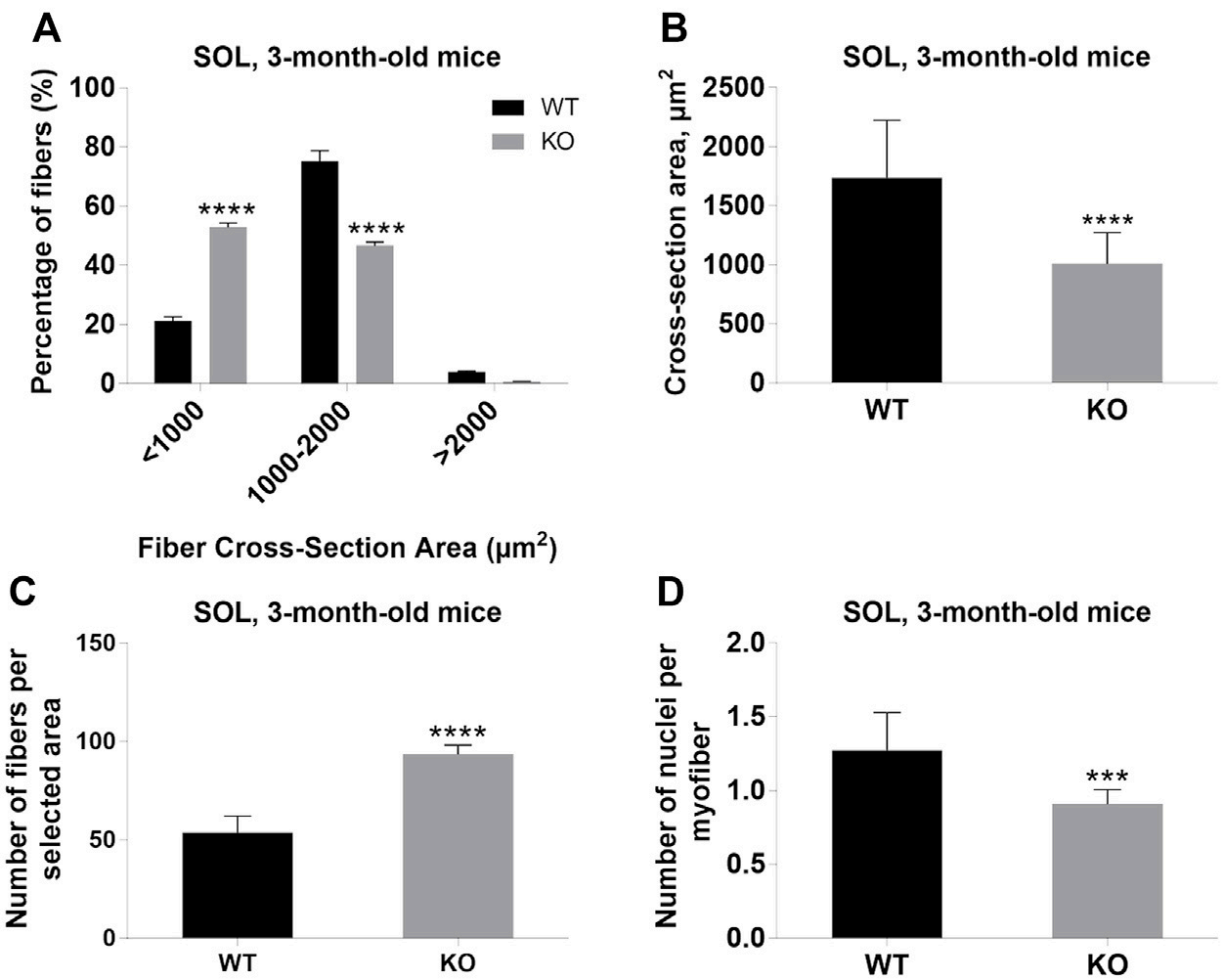
Analysis of soleus (SOL) muscles of 3-month-old WT and KO mice. **(A)**, Size distribution of muscle fibers. **(B)**, Cross-section area. **(C)**, The average number of myofibers per selected area. **(D),** The number of nuclei per myofiber. Data are expressed as mean ± SD, t-test, ***p ≤ 0.001, ****p < 0.0001; vs. control.

**FIGURE 4:**
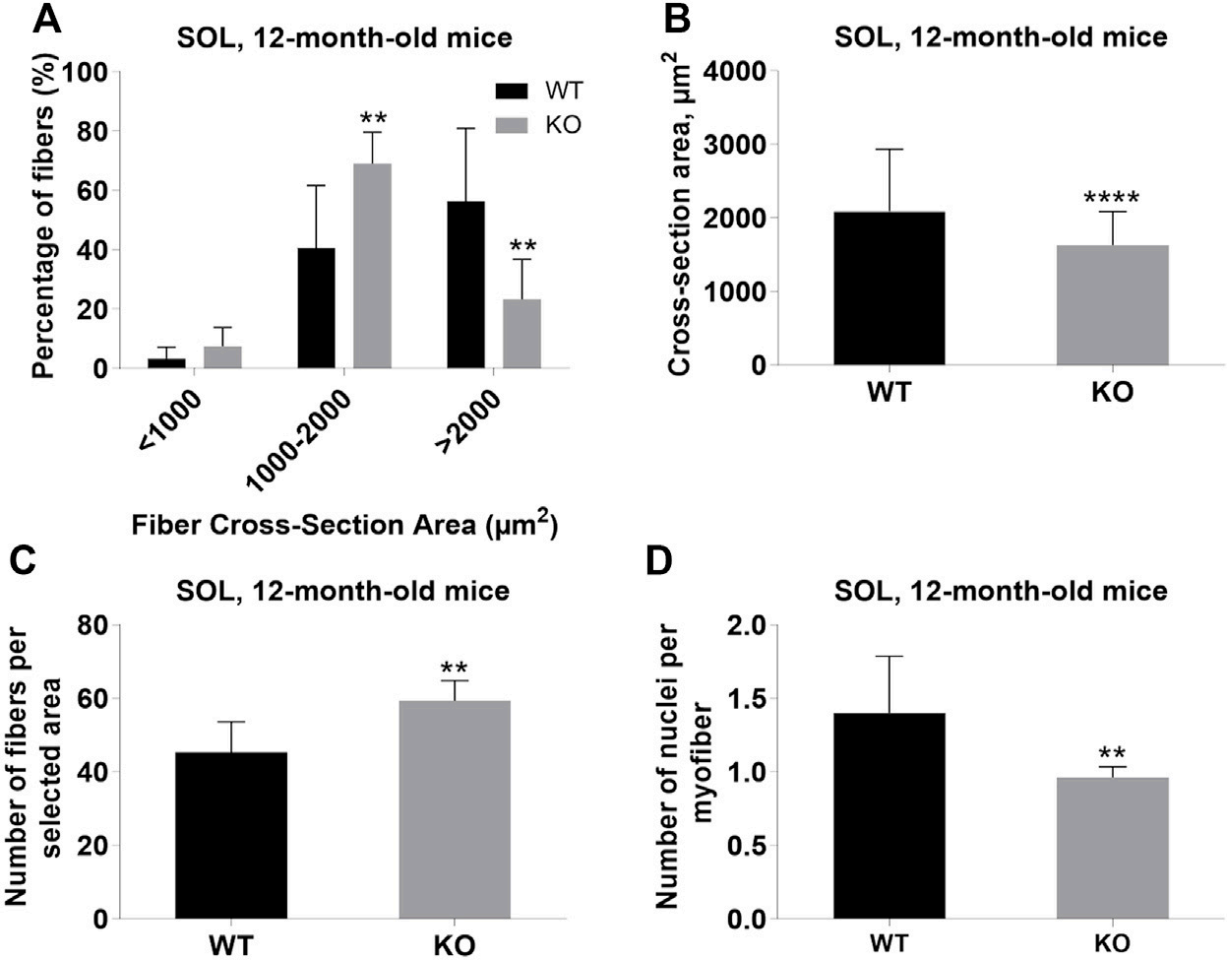
Analysis of soleus (SOL) muscles of 12-month-old WT and KO mice. **(A),** Size distribution of muscle fibers. **(B)**, Cross-section area of hindlimb muscles. **(C)**, The average number of myofibers per selected area. **(D)**, Number of nuclei per myofiber. Data are expressed as mean ± SD, t-test, **p ≤ 0.01, ****p < 0.0001; vs. control.

**FIGURE 5:**
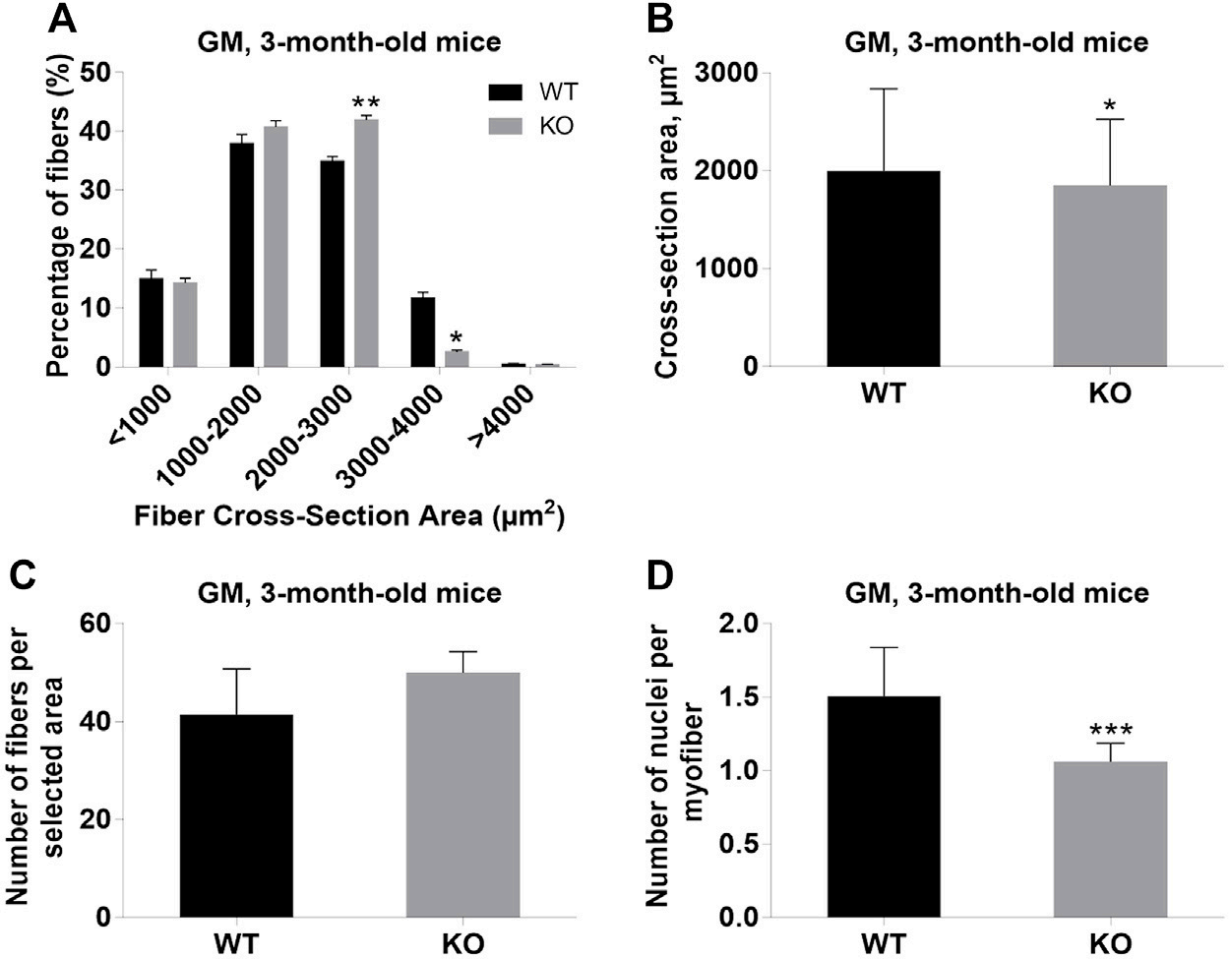
Analysis of gastrocnemius medialis (GM) muscles of 3-month-old WT and KO mice. **(A)**, Size distribution of muscle fibers. **(B)**. Cross-section area of hindlimb muscles. **(C)**, The average number of myofibers per selected area. **(D)**, Number of nuclei per myofiber. Data are expressed as mean ± SD, t-test, *p ≤ 0.05; **p ≤ 0.01, ***p ≤ 0.001; vs. control.

**FIGURE 6:**
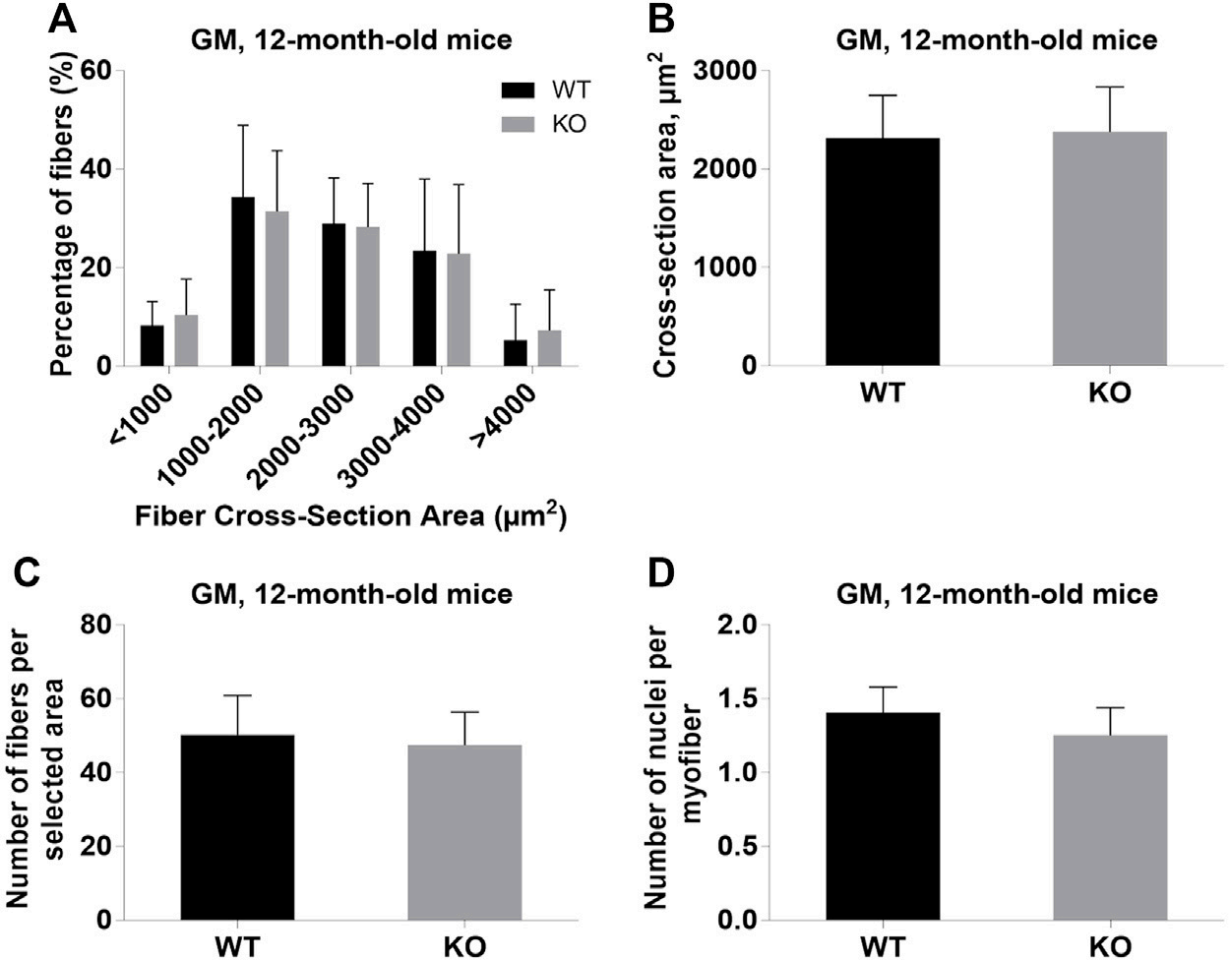
Analysis of gastrocnemius medialis (GM) muscles of 12-month-old WT and KO mice. **(A),** Size distribution of muscle fibers. **(B)**, Cross-section area of hindlimb muscles. **(C)**, The average number of myofibers per selected area. **(D)**, Number of nuclei per myofiber. Data are expressed as mean ± SD.

Next, we performed a detailed analysis of the fiber size of the hindlimb muscles at P0, 3-months, and 12-months of age. Analysis of hematoxylin/eosin staining of cross-sections of P0 hindlimb muscle revealed variability in the myofiber size (Figure 2A). Quantification revealed that KO muscles contained a higher amount of small fibers (cross-section area (CSA) less than 100 µm^2^) with a concomitant decrease in the amount of larger fibers (cross-section area more than 100 µm^2^) (Figure 2B). The average fiber CSA was significantly smaller for KO muscles (140 µm^2^ in WT vs. 110 µm^2^ in SV mice) (Figure 2C). This was accompanied by a higher number of myofibers per selected area in KO muscles (Figure 2D). Analogous results were obtained for SOL muscles isolated from 3- and 12-month-old mice (3-months of age: 1750 µm^2^ in WT vs. 1000 µm^2^ for KO; 12-months of age: 2000 µm^2^ in WT vs. 1600 µm^2^ in KO) (Figure 3, Figure 4, respectively). These data indicate that the increase in the muscle:body mass ratio of KO SOL muscles results from a significantly larger amount of small fibers and not from an increase in overall myofiber size. A similar tendency was observed for GM muscles of 3-month-old mice (Figure 5). However, the parameters obtained for WT and KO GM muscles derived from 12-month-old mice did not differ significantly (Figure 6). Thus, the biggest differences between WT and KO muscles were seen in newborn animals (where the process of muscle maturation has not been completed), as well as slow-twitch SOL muscles during the entire examined animal lifespan.

### Loss of Myosin VI Affects cAMP Level

**FIGURE 7:**
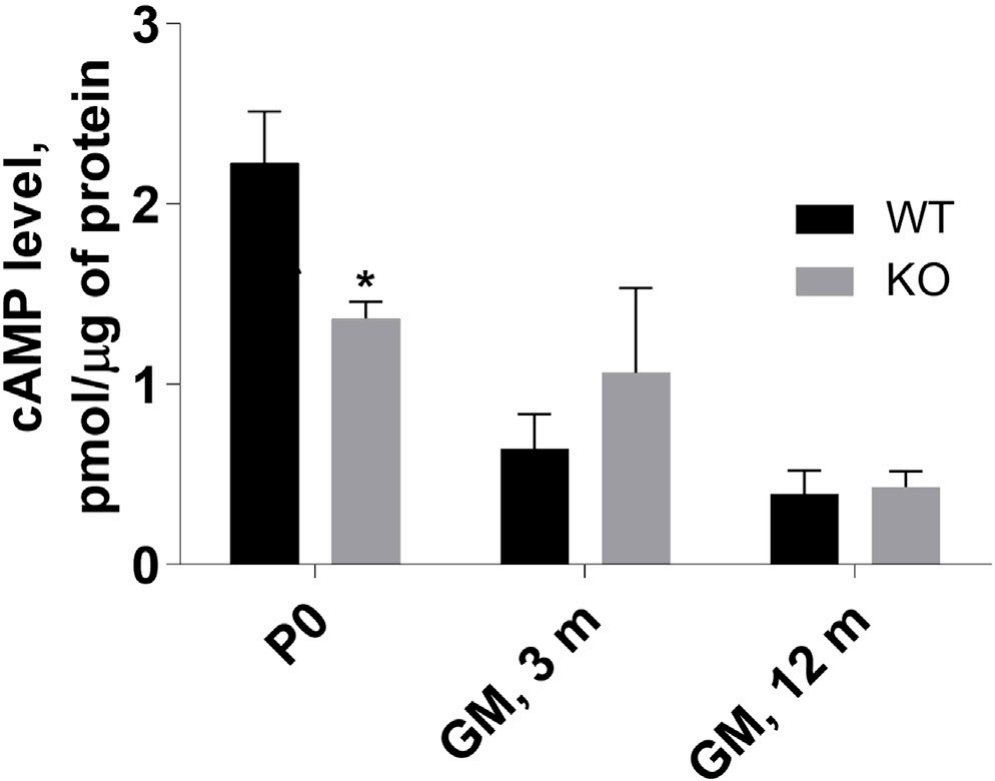
Assessment of cAMP level in P0 hindlimb as well as in 3- and 12-months gastrocnemius medialis (GM) muscle. The levels were evaluated in muscle homogenates as described in Materials and methods. Data are expressed as mean ± SD, t-test, *p ≤ 0.05; vs. control.

Our previous results demonstrated an increase in cAMP level in MVI-depleted C2C12 undifferentiated myoblasts, indicating the involvement of MVI in cAMP signaling (Karolczak et al., 2015). Evaluation of cAMP level in P0 hindlimb muscles as well as in adult GM muscle revealed that the level was significantly decreased in P0 muscle, increased in 3-month-old GM (though without a statistical relevance), and unchanged in 12-month-old muscles (Figure 7). These results indicate that the differences in the level of this nucleotide between WT and KO muscles vary during the lifespan, suggesting that the observed variability in the effects of lack of MVI on cAMP signaling may depend on age.

### Loss of Myosin VI and Expression of Proteins of cAMP Signaling

**FIGURE 8:**
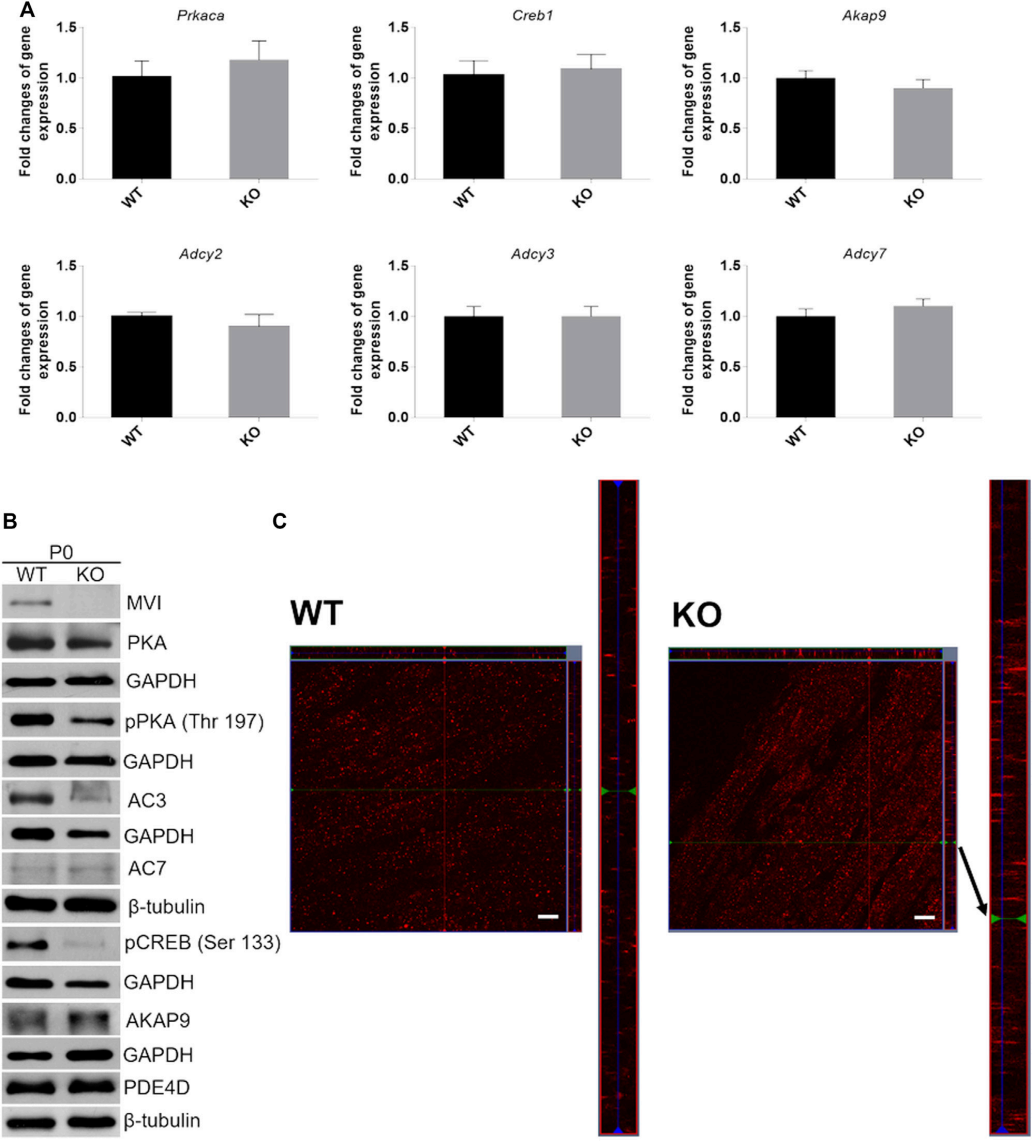
Analysis of cAMP/PKA signaling pathway in hindlimb muscles isolated from P0 mice. **(A)**, RT-qPCR analysis of mRNA levels of key genes involved in cAMP/PKA signaling (see Figure 1B). The data are expressed as mean ± SD, t-test, *n* = 8, *p ≤ 0.05 vs. control. **(B)**, Representative Western blot of key proteins involved in cAMP/PKA pathway. GAPDH or *β*-tubulin served as the internal loading control. **(C)**, Localization of AKAP9 (red staining) in muscles of WT (left panels) and KO (right panels) mice. Bars, 10 µm.

Next, we evaluated the effects of lack of MVI on the expression of genes engaged in cAMP signaling (see Figure 1B) in muscles isolated from newborn mice. We examined both mRNA and protein levels (Figure 8 and Supplementary Figure S2). Analysis of the transcript level revealed that in P0 samples expression of genes encoding PKA (*Prkaca*), CREB (*Creb1*), AKAP9 (*Akap9*) as well as three isoforms of adenylate kinase, AC2, AC3, and AC7 (*Adcy2, Adcy3,* and *Adcy7,* respectively) that are present in skeletal muscle was not affected by the absence of MVI (Figure 8A). Analysis of the protein level showed that loss of MVI did not significantly affect the levels of PKA, AC7, AKAP9, and PDE4. However, it caused a decrease in the levels of AC3, as well as of active forms of CREB (pCREB) and PKA (pPKA) (Figure 8B; densitometric analyses are presented in Supplementary Figure S2).

**FIGURE 9:**
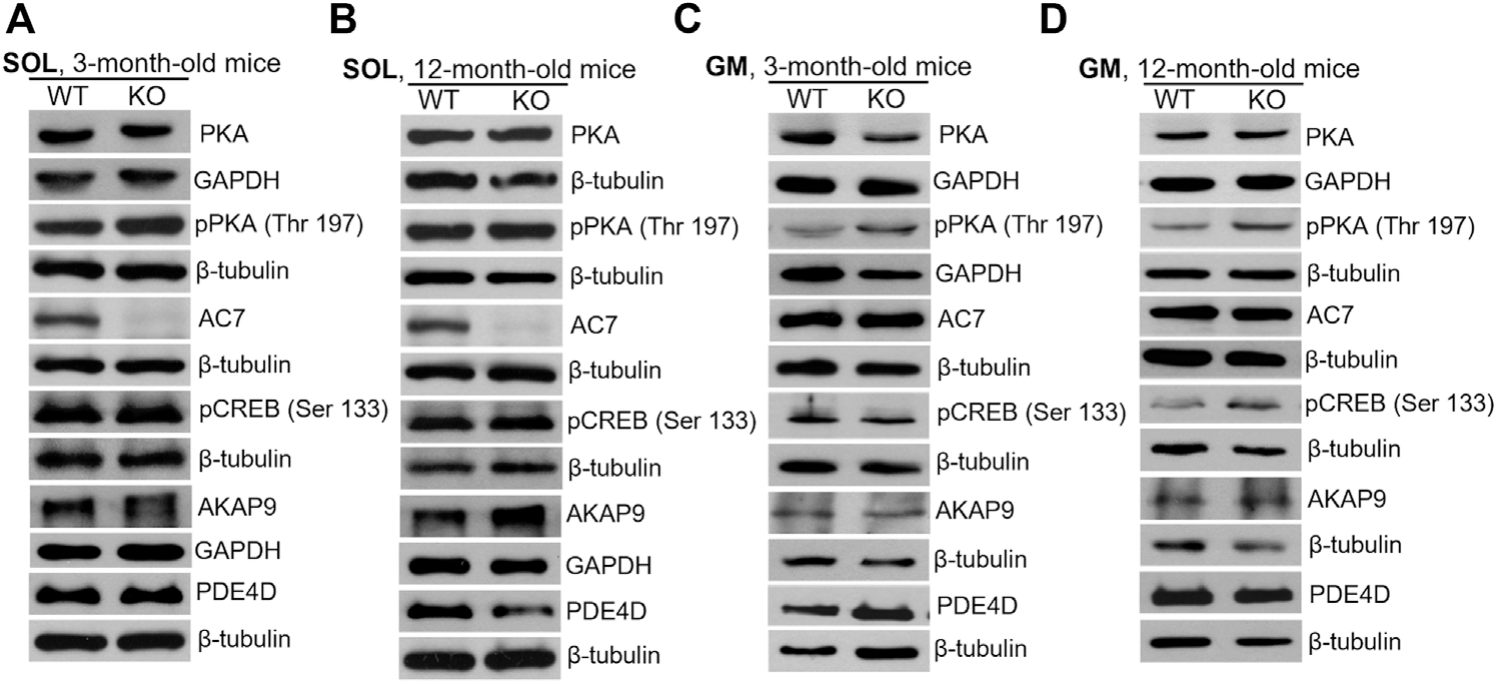
Changes in the levels of key proteins involved in cAMP/PKA pathway in soleus (SOL; **(A)** and **(B)**) and gastrocnemius medialis (GM; **(C)** and **(D)**) muscles isolated from 3- **(A)** and **(C)** and 12-month-old **(B)** and **(D)**) WT and KO mice. GAPDH or *β*-tubulin served as the internal loading control.

Similar analyses of the protein level were performed for SOL and GM muscles isolated from 3-months and 12-month-old mice (Figure 9; densitometric analyses are presented in Supplementary Figures S3–S6). Unlike newborns, there was practically no difference in the levels of the examined proteins in WT and KO muscles except of AC7, the level of which was significantly decreased in 3- and 12-month-old SOL muscles (Figures 9A,B, and Supplementary Figures S3,S4). Also, a decreased AKAP9 content, and increased PDE4D content, were observed for GM muscle of 3- and 12-month-old mice, respectively. These data suggest that MVI is mainly involved in the PKA signaling pathway in immature, newborn muscles but not in muscles of adult animals, where other pathway(s) could be activated.

### Analysis of AKAP9 Localization in Muscles of Newborn Mice

Since AKAP9 is known to interact with MVI (Karolczak et al., 2015), we examined whether the lack of this molecular motor could affect its distribution (Figure 8C). Anti-AKAP9 immunostaining of longitudinal sections of the WT P0 muscle revealed that this scaffold protein localized to the elongated puncta and was dispersed uniformly throughout the fiber (Figure 8C, left panels). While the overall localization pattern was similar in KO muscle, the puncta size was more variable as both very small and very long structures were present (Figure 8C, right panels).

**FIGURE 10:**
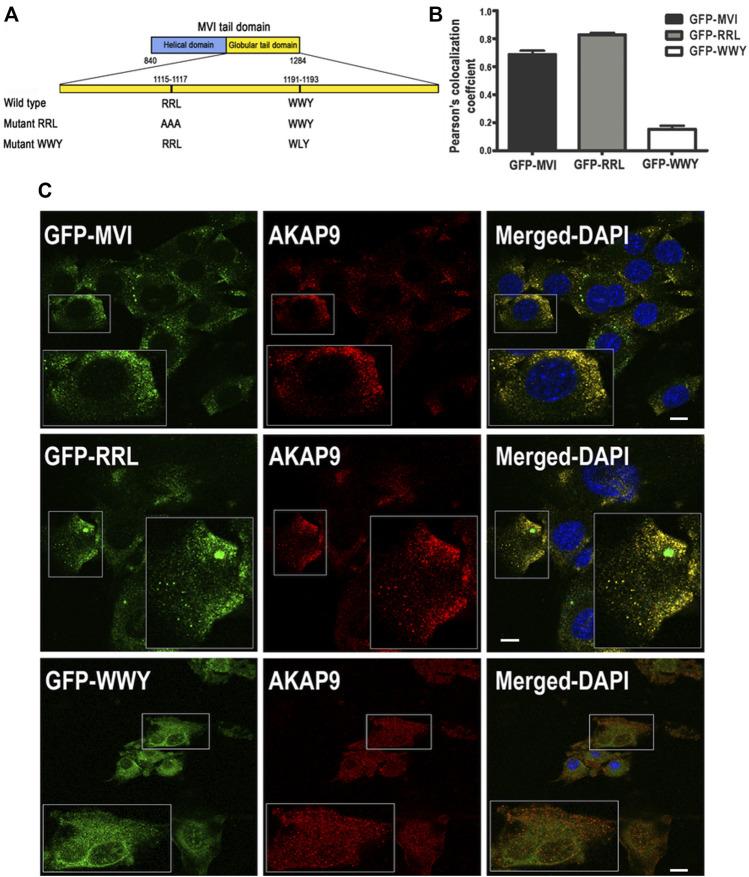
Interaction of myosin VI with AKAP9. **(A)**, depiction of partner interaction sites within MVI globular tail domain and its mutants (RRL and WWY). **(B)**, values of Pearsons’s coefficient characterizing the interaction of AKAP9 with GFP-tagged MVI and its mutants. **(C)**, Co-localization of AKAP9 (in red, probed with anti-AKAP9 antibody) with GFP-tagged MVI and its mutants (in green) in murine C2C12 myoblasts. Insets, ∼2x magnification of the marked area. Bars, 10 µm. Further details in Materials and methods.

We next attempted to characterize the nature of the MVI-AKAP9 interaction. For this, we used GFP-tagged MVI tail domains with mutations within the regions involved in the partner binding where RRL (involved in electrostatic interactions) was mutated to AAA, and WWY (involved in hydrophobic interactions) was mutated to WLY (Figure 10A (Tumbarello et al., 2012)). Thus, the RRL mutant can interact with cargo only *via* the unmodified WWY motif, and the WWY mutant can interact only *via* the unmodified RRL motif. C2C12 myoblasts were transfected with GFP-MVI tail domain (WT) and its RRL and WWY mutants followed by immunostaining with anti-AKAP9 antibody (Figures 10B,C). WT and RRL mutant tails co-localized with AKAP in puncta dispersed throughout the cell with Pearson’s correlation coefficient ranging from ∼0.68 in WT to ∼0.89 in the mutant, respectively. However, there was practically no co-localization between AKAP9 and WWY mutant as the Pearson’s correlation coefficient was only ∼0.15. Thus, these data indicate that AKAP9 interacts with the MVI globular tail domain through the WWY binding site, ergo through hydrophobic interactions.

### Distribution of CREB in P0 Muscle

**FIGURE 11:**
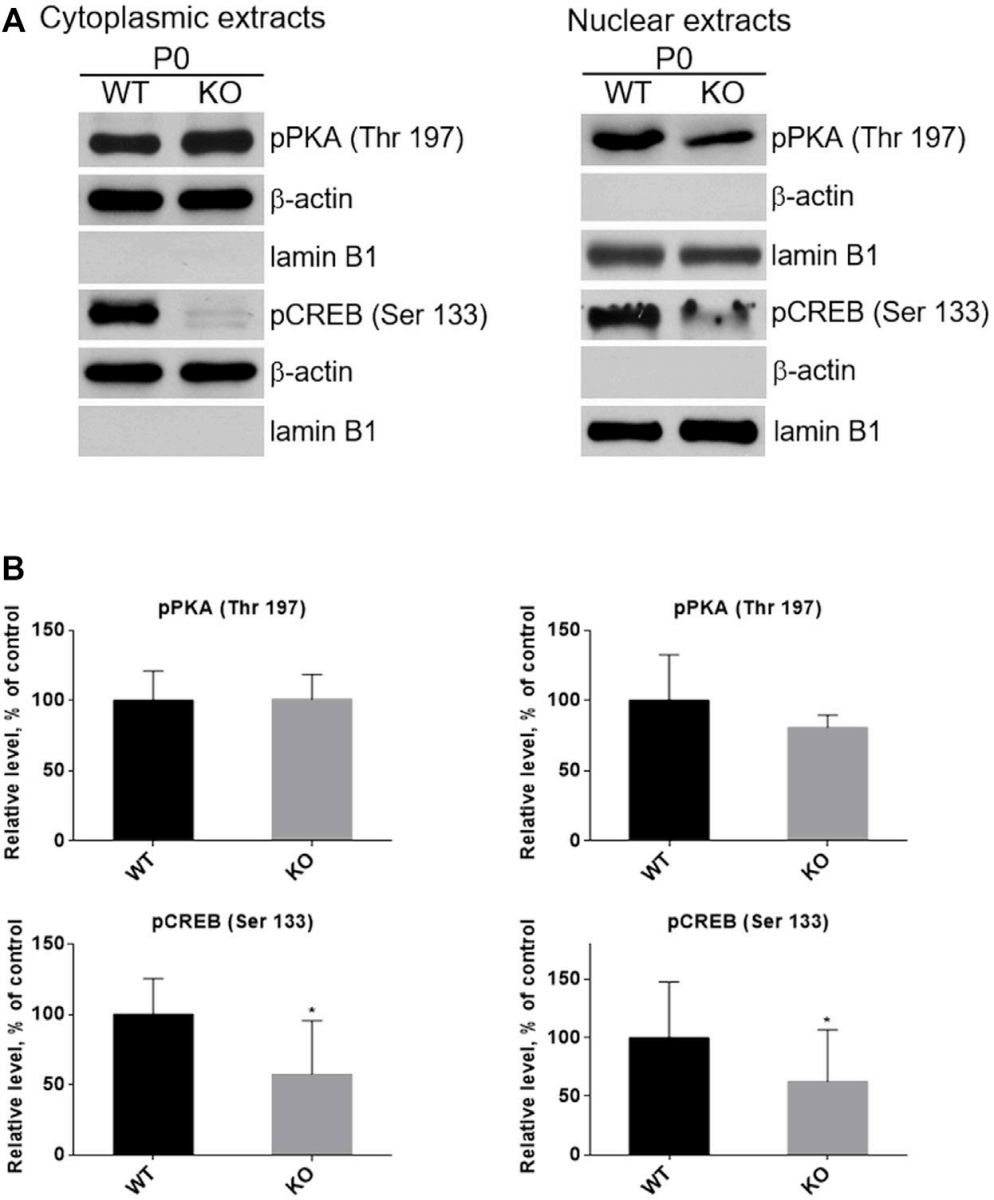
Examination of the presence of active forms of PKA (pPKA) and CREB (pCREB) in subcellular compartments. **(A)**, Detection of pPKA and pCREB proteins in the cytoplasmic (left panel) and nucleoplasmic (right panel) fractions of hindlimb muscles homogenates of P0 WT and KO mice. *β*-actin and lamin B1 served as the internal loading control and fraction purity. **(B)**, Densitometric analysis of the levels pPKA and pCREB in the examined fractions. Data are expressed as mean ± SD, t-test, *p ≤ 0.05 vs. control.

CREB, the cAMP/PKA dependent transcription factor (Figure 1B), plays important role in skeletal muscle function and myogenesis (Carlezon et al., 2005; Altarejos and Montminy, 2011). The observations that the levels of cAMP and active forms of CREB (pCREB), phosphorylated at Ser133, as well as of PKA (pPKA), phosphorylated at Thr197, are significantly lower in P0 KO muscle (and not in muscles of adult animals) suggest that loss of MVI may not only impact the activity of these proteins but also their subcellular localization in unmatured, P0, muscles. To test this, we separated P0 muscle homogenates into cytoplasmic and nuclear fractions, and then evaluated the levels of pPKA and pCREB in each fraction (Figures 11A,B). Cytoplasmic levels of pPKA were similar in WT and KO muscle samples, but the level of cytoplasmic pCREB was significantly lower in KO muscles. Similar results were found in the nuclear fraction, as the amount of pCREB was lower in the nuclei of KO muscles as was the amount of pPKA, though the decrease of the pPKA level did not reach statistical significance. These data indicate that lack of MVI affects cellular localization of pCREB and, to a lesser extent, pPKA.

## Discussion

The current study builds on our previous findings showing that AKAP9, a regulator of PKA activity, interacts in myogenic cells with the MVI cargo binding domain (which is also directly phosphorylated by PKA). Here, we examined the role of MVI in cAMP/PKA signaling in skeletal muscle. We demonstrate that lack of MVI affects muscle morphology and cAMP/PKA signaling in murine hindlimb muscles, in particular in tissues derived from newborn animals, indicating the involvement of MVI in the age-dependent regulation of PKA-dependent muscle metabolism.

Our decision to evaluate the hindlimb muscles of MVI knockout mouse model (*SV*, KO) at different ages (*i.e.* newborn - P0 and adult 3- and 12-month-old mice) was of particular importance as adult KO mice are known to be smaller and hyperactive with respect to control, heterozygous animals (Deol and Green, 1966; Hegan et al., 2015). This implies that any changes observed in adult and aged mice could be related to the hyperactive phenotype and not to changes associated with loss of MVI *per se*. Our observation that newborn KO mice are smaller indicate that lack of MVI could greatly contribute to muscle development. Interestingly, changes in the muscle/body mass ratio, which indicate muscle enlargement, are not due to an increase in fiber size but rather to an increased abundance of small fibers; this tendency is also visible in muscles of adult mice. It is noteworthy that Hegan et al. (2015) showed that the hearts of adult *SV* animals are hypertrophic. Interestingly, symptoms of hypertrophic cardiomyopathy have been reported in adult patients with a point mutation within *MYO6* (Mohiddin et al., 2004).

Muscle mass increase could result from increased myofiber size (fiber hypertrophy) or from the formation of new myofibers (fiber hyperplasia) (Fernandez et al., 2002). The observation that the average CSA of KO myofibers of all the examined muscles was reduced while the average number of fibers per selected area was increased with a shift into a higher number of smaller fibers indicates that KO muscles are hyperplastic and not hypertrophic. This was unexpected as our previous observations on myoblasts showed that a lack MVI results in the formation of aberrant, thick myotubes with a spindle-like morphology (Lehka et al., 2020). Furthermore, the reduced number of myonuclei per myofiber in KO adult muscles indicates the presence of alterations in the organization of the myonuclear domain (increased amount of cytoplasm per nucleus). We hypothesize that these morphological changes might be partially due to alterations in the cAMP/PKA signaling pathway, which is well-known to be involved in muscle development and metabolism (Taylor et al., 2013) and has already been shown to be associated with MVI in myogenic cells (Karolczak et al., 2015).

Numerous studies have shown that modulating the cAMP/PKA signaling pathway can have adaptive effects on skeletal muscles, by either increasing or decreasing myofiber size, and promoting fiber-type transitions to glycolytic fibers (Hinkle et al., 2003; Spurlock et al., 2006; Ryall et al., 2008). cAMP mediates the cell response to multiple hormones and neurotransmitters, and regulates a plethora of cellular processes, including metabolism, gene expression, cell growth, and division of myogenic cells (Stork and Schmitt, 2002). In rodent adult skeletal muscle, the most abundant forms of adenylyl cyclases (involved in the synthesis of cAMP) are AC2, AC3, AC7, and AC9 (Torgan et al., 1996; Suzuki et al., 1998). We did not observe a difference in the levels of transcipts of the genes encoding these enzymes. However, on the protein level, the amount of AC3 was substantially decreased in P0 KO hindlimb muscles. This was accompanied by a decrease in cAMP level, suggesting a correlation between lack of MVI and decrease in cAMP, and the resulted aberrations cAMP signaling in immature muscle. In the homogenates of adult SOL and GM muscles, AC3 was not detectable but we were able to detect AC7 and found it to be significantly decreased in adult KO SOL muscle but not in GM muscle. In contrast, the levels of PDE4D, a member of phosphodiesterase (PDE) family that plays important role in the degradation of cAMP, thus determining the amplitude and duration of cAMP signals in response to different stimuli (Houslay, 2010), was significantly increased in KO GM muscles of aged muscles, suggesting a difference in the effects of lack of MVI dependent on the myofiber type. We cannot exclude the possibility that in 3- and 12-month-old SOL and GM muscles, where we did not observe a correlation between the presence of MVI and the level of cAMP, there are aberrations in the synthesis of other AC and/or PDE isoforms that could affect the levels of cAMP and cAMP/PKA pathways.

In P0 muscles we observed a significant decrease in PKA activity, the main effector of cAMP, that upon binding cAMP, phosphorylates in a spatiotemporal manner a specific subset of downstream targets, including MVI (Terrin et al., 2012; Karolczak et al., 2015). Interestingly, we did not observe a decrease of PKA activity in SOL and GM muscles of 3- and 12-month-old mice, further suggesting that the cAMP/PKA pathway depends on the presence of MVI mainly in immature P0 muscles. In line with this, we observed that the activity of CREB, a cAMP-dependent transcription factor that plays a key role in the differentiation of embryonic skeletal muscle progenitors and functions in adult skeletal muscle by activation of a number of genes (Figure 1B) (Stewart et al., 2011), was only affected in P0 muscles. We also observed that nuclear localization of active CREB (phosphorylated at Ser133 by PKA) in P0 muscles also depends on the presence of MVI, suggesting that MVI, known to shuttle between the cytoplasm and nucleus (Majewski et al., 2018), could be involved in its delivery to the nucleus of the myofibers that still undergo maturation/remodeling.

Based on our earlier observations on myogenic cells (Karolczak et al., 2015), we expected that lack of MVI would result in an increase in the level of MVI binding partner, AKAP9, which is a member of the family of A kinase–anchoring proteins. AKAP9 is important for compartmentalization of constituents of cAMP/PKA signaling, thus providing spatiotemporal regulation of this pathway, also in myogenic cells and muscle tissue (Figure 1B; Wong and Scott, 2004). However, we did not observe statistically significant difference in the AKAP9 level neither in P0 nor in 3- and 12-month-old KO muscles (except for a decrease in GM muscle of adult KO mice). Despite that we did not see a change in the level of AKAP9, we observed that lack of MVI affected the localization of AKAP9 in P0 muscle as abnormal elongated aggregate-like structures were visible in KO muscles. This data suggests that MVI could be involved in the organization of myofiber compartment(s) associated with AKAP9. Also, we demonstrated that AKAP9 interacts with MVI globular cargo binding domain AKAP9 *via* the WWY motif, i.e. *via* hydrophobic interactions. It has been shown that proteins involved in endocytosis such as Dab-2 and Tom1 also interact with MVI *via* WWY binding site (Tumbarello et al., 2013; Magistrati and Polo, 2021). It is noteworthy that AKAP9 was found to be present within Rab5-coated endocytotic vesicles associated with MVI (Karolczak et al., 2015).

### Conclusion

Our data show that the association of MVI with the cAMP/PKA signaling pathway is important for two major reasons. Firstly, the involvement of MVI in this pathway seems to be age-dependent as the biggest differences caused by MVI depletion were visible in hindlimb muscles of newborn, immature muscles; this was shown on the morphological and protein synthesis levels. This is consistent with findings that cAMP/PKA signaling plays important roles in myogenesis and muscle regeneration (Stork and Schmitt, 2002; Taylor et al., 2013). Secondly, involvement of MVI in cAMP/PKA signaling seems to depend on myofiber type as we observed differences in effects of lack of MVI between SOL and GM muscles, with slow-twitch SOL muscle being more sensitive to lack of this molecular motor than fast-twitch GM muscle. This observation indicates a previously unknown role of MVI in muscle oxidative metabolism. Also, it is plausible that the lack of MVI in mature muscles caused aberrations in other pathways that are associated with muscle metabolism. Further studies are needed to understand the mechanisms behind these differences and to reveal whether and how lack of MVI affects muscle contractions.

## Data Availability

The original contributions presented in the study are included in the article/Supplementary Material, further inquiries can be directed to the corresponding author.
