# Supplementary material for: Loss of Unconventional Myosin VI Affects cAMP/PKA Signaling in Hindlimb Skeletal Muscle in an Age-Dependent Manner
Source: Front Physiol. 2022 Jun 28;13:933963. doi: 10.3389/fphys.2022.933963 (PMC9273875; doi:10.3389/fphys.2022.933963)
Supplement: Supplementary file 1 [file DataSheet1.PDF]

Supplementary Material

Supplementary Table 1

Hindlimb muscle mass (in mg) of WT and KO mice

Age	GM		SOL		TA		EDL	
	WT	KO	WT	KO	WT	KO	WT	KO
3-month-old	145.7±18.3	115.1±17.6	9.1±1.1	8.0±1.5	47.5±8.7	34.0±8.5	11.7±2.6	7.9±1.5
12-month-old	158.4±16.4	123.5±11.1	9.8±1.3	9.7±1.3	52.8±8.2	39.7±9.7	12.7±3.4	10.9±2.9

Muscles isolated from 3-month-old and 12-month-old WT and KO male mice were weighed. GM, gastrocnemius medialis, SOL, soleus, TA, tibialis anterior and EDL, extensor digitorum longum. Values are presented as mean ± SD. At least 14 animals of each group were used for the analysis.

Supplementary Figure 1

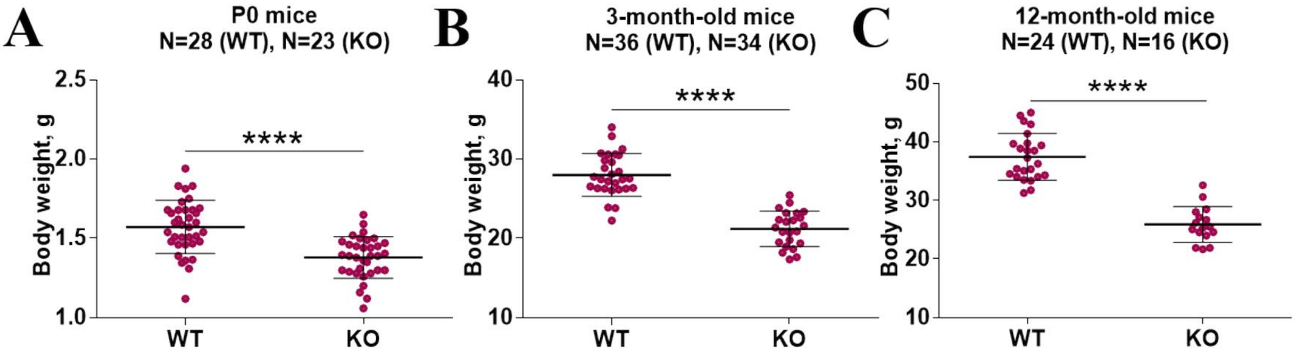

Supplementary Figure 1. Analysis of body mass of WT and KO male mice. **(A)**, newborn animals; **(B)**, 3-month-old animals; **(C)**, 12-month-old animals. Data are expressed as mean±SD, t-test, **** $p<0.0001$; versus control.

Supplementary Figure 2

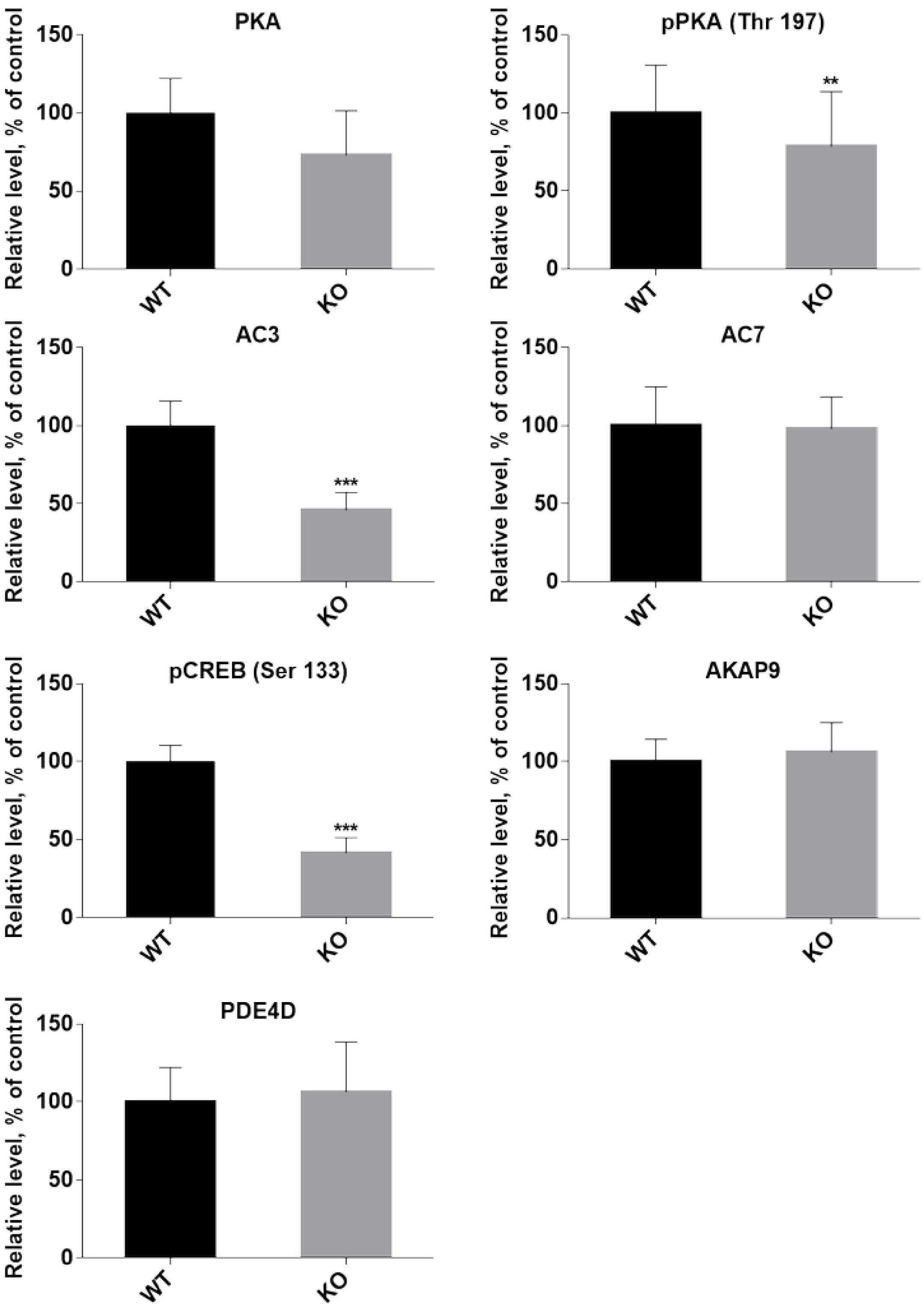

Supplementary Figure 2. Densitometry analysis of key proteins involved in cAMP/PKA pathway in homogenates of P0 hindlimb muscle (representative Western blots are presented in Figure 8B). Analysis was performed for at least three muscle homogenates of each examined group. Data are expressed as mean±SD, t-test, ** $p \leq 0.01$, ***$p \leq 0.001$; vs control.

Supplementary Figure 3

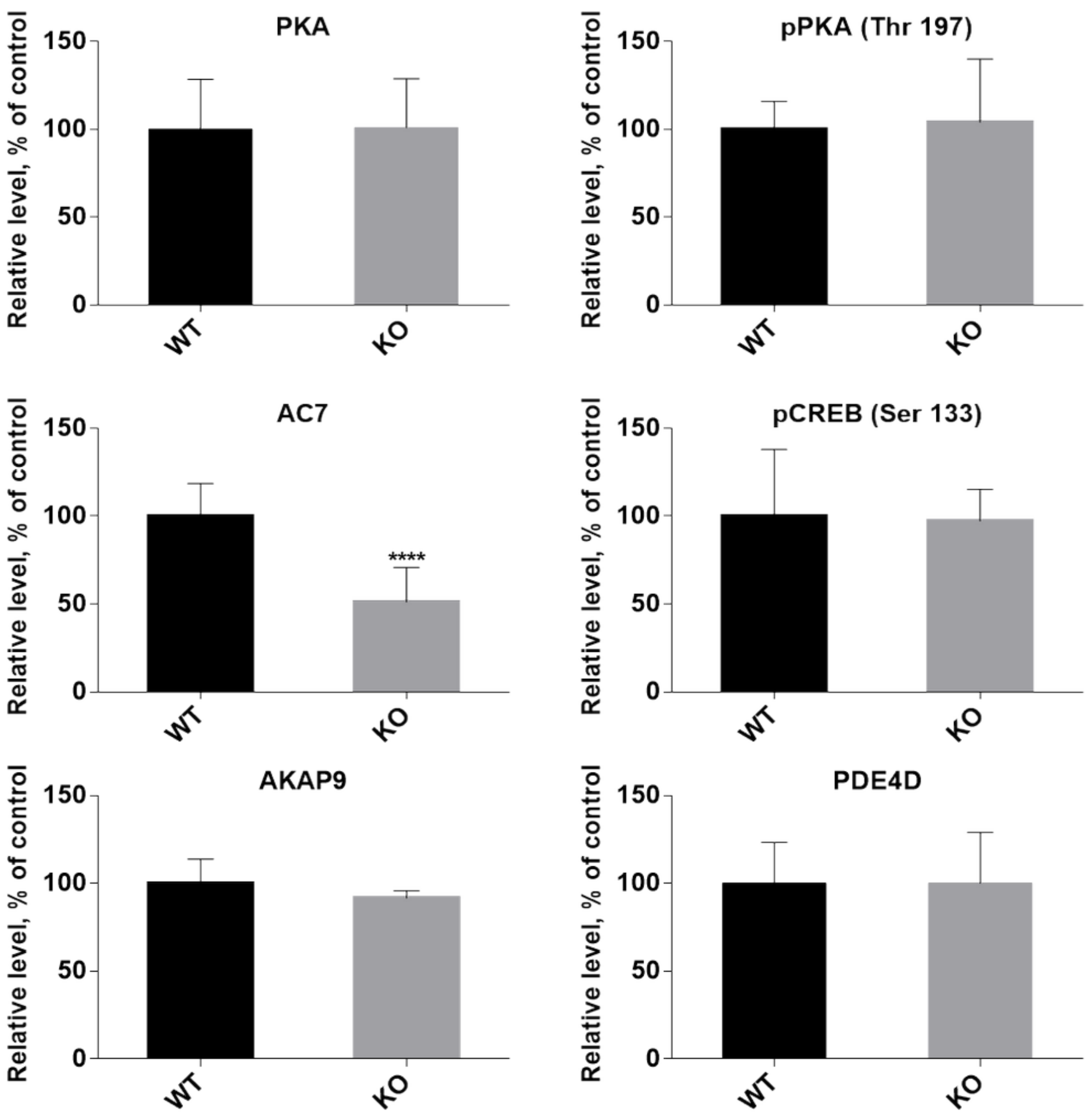

Supplementary Figure 3. Densitometry analysis of key proteins involved in cAMP/PKA pathway in 3-month-old SOL muscle (representative Western blots are presented in Figure 9A). Analysis was performed for at least three muscle homogenates of each examined group. The data are expressed as mean±SD, t-test, ****p≤ 0.0001 vs control.

Supplementary Figure 4

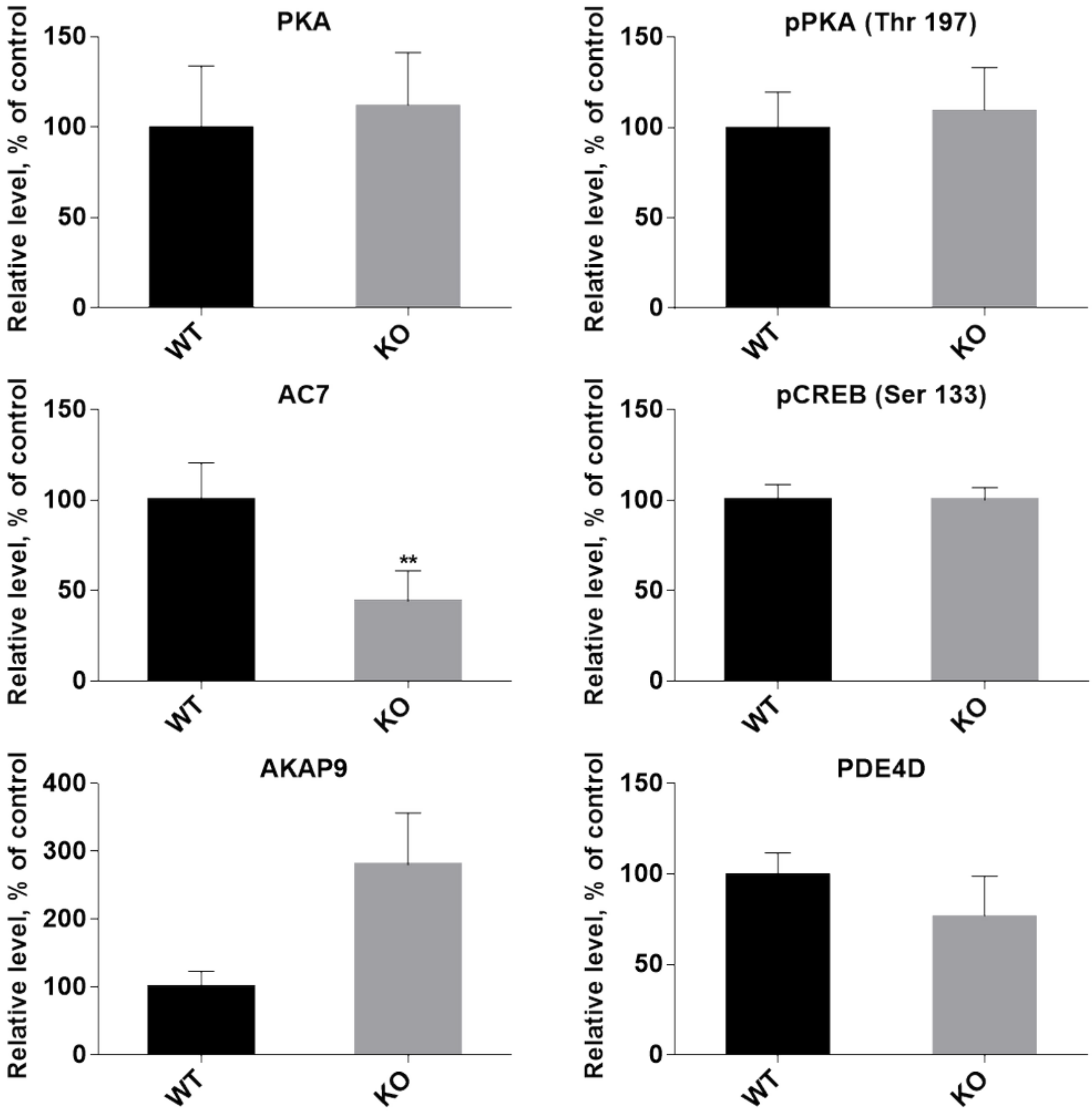

Supplementary Figure 4. Densitometry analysis of key proteins involved in cAMP/PKA pathway in 12-month-old SOL muscle (representative Western blots are presented in Figure 9B). Analysis was performed for at least three muscle homogenates of each examined group. The data are expressed as mean±SD, t-test, ** p≤ 0.01; vs control.

Supplementary Figure 5

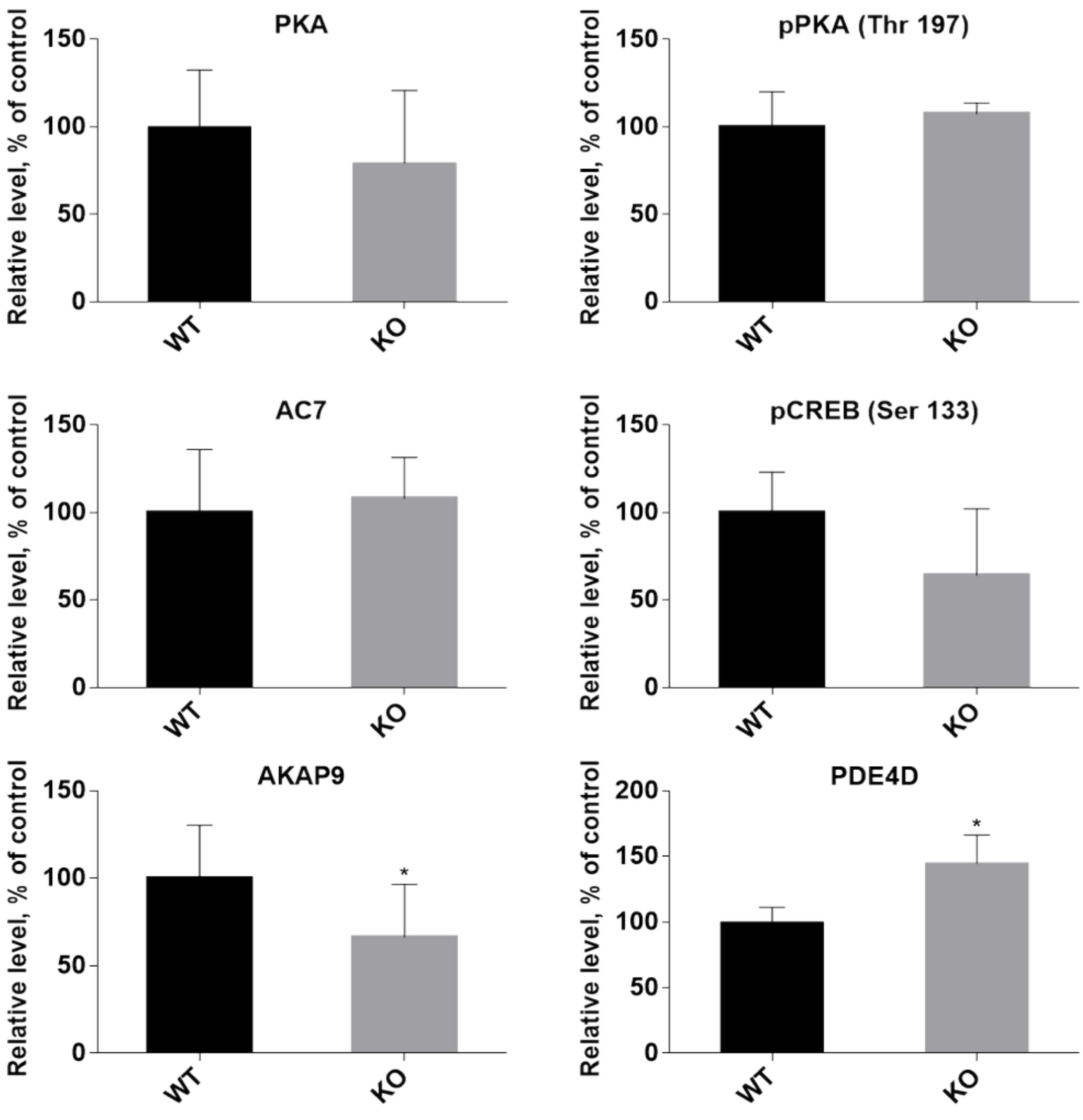

Supplementary Figure 5. Densitometry analysis of key proteins involved in cAMP/PKA pathway in 3-month-old GM muscle (representative Western blots are presented in Figure 9C). Analysis was performed for at least three muscle homogenates of each examined group. The data are expressed as mean±SD, t-test, * $p \leq 0.05$; vs control.

Supplementary Figure 6

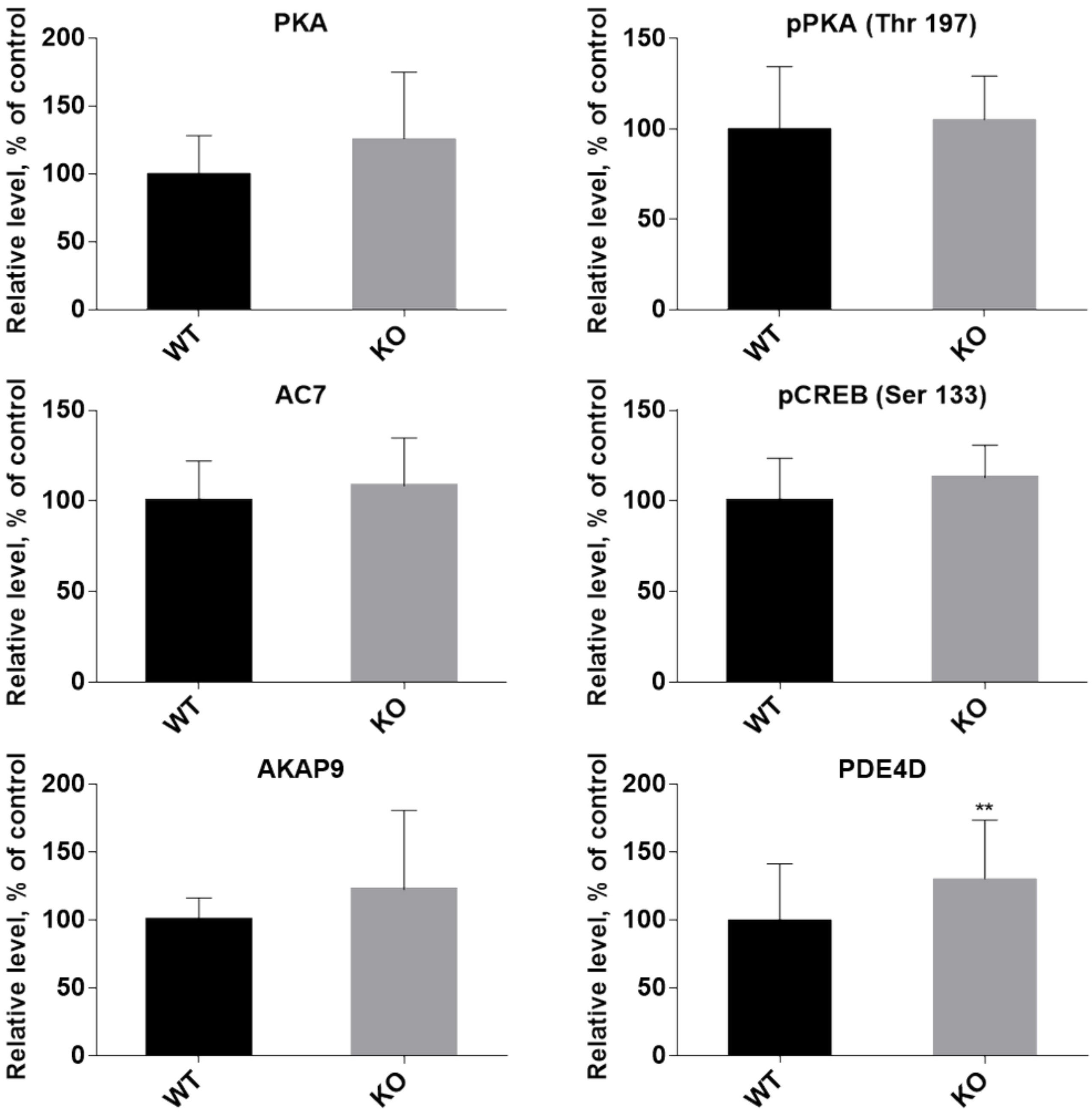

Supplementary Figure 6. Densitometry analysis of key proteins involved in cAMP/PKA pathway in 12-month-old GM muscle (representative Western blots are presented in Figure 9D). Analysis was performed for at least three muscle homogenates of each examined group. The data are expressed as mean±SD, t-test, ** p≤ 0.01 vs control.
